# Functional phenomics for improved climate resilience in Nordic agriculture

**DOI:** 10.1093/jxb/erac246

**Published:** 2022-06-21

**Authors:** Thomas Roitsch, Kristiina Himanen, Aakash Chawade, Laura Jaakola, Ajit Nehe, Erik Alexandersson

**Affiliations:** Department of Plant and Environmental Sciences, University of Copenhagen, Denmark; Department of Adaptive Biotechnologies, Global Change Research Institute, Czech Academy of Sciences, Brno, Czechia; National Plant Phenotyping Infrastructure, HiLIFE, University of Helsinki, Finland; Organismal and Evolutionary Biology Research Program, Viikki Plant Science Centre, Faculty of Biological and Environmental Sciences, University of Helsinki, Finland; Department of Plant Breeding, Swedish University of Agricultural Sciences, Lomma, Sweden; Climate laboratory Holt, Department of Arctic and Marine Biology, UiT the Arctic University of Norway, Tromsø, Norway; NIBIO, Norwegian Institute of Bioeconomy Research, Ås, Norway; Department of Plant Breeding, Swedish University of Agricultural Sciences, Lomma, Sweden; Department of Plant Protection Biology, Swedish University of Agricultural Sciences, Lomma, Sweden; National Research Council, Italy

**Keywords:** Arctic, climate change, crop phenotyping, functional phenomics, Nordic agriculture, wild crops

## Abstract

The five Nordic countries span the most northern region for field cultivation in the world. This presents challenges *per se*, with short growing seasons, long days, and a need for frost tolerance. Climate change has additionally increased risks for micro-droughts and water logging, as well as pathogens and pests expanding northwards. Thus, Nordic agriculture demands crops that are adapted to the specific Nordic growth conditions and future climate scenarios. A focus on crop varieties and traits important to Nordic agriculture, including the unique resource of nutritious wild crops, can meet these needs. In fact, with a future longer growing season due to climate change, the region could contribute proportionally more to global agricultural production. This also applies to other northern regions, including the Arctic. To address current growth conditions, mitigate impacts of climate change, and meet market demands, the adaptive capacity of crops that both perform well in northern latitudes and are more climate resilient has to be increased, and better crop management systems need to be built. This requires functional phenomics approaches that integrate versatile high-throughput phenotyping, physiology, and bioinformatics. This review stresses key target traits, the opportunities of latitudinal studies, and infrastructure needs for phenotyping to support Nordic agriculture.

## Teaser

Functional phenomics can guide targeted breeding and precision agriculture to accelerate local adaptation to make Nordic farming more efficient and resilient, also within future climate scenarios.

## Introduction

### The role of functional phenomics to meet current needs and future challenges in Nordic agriculture

Agriculture in the Nordic countries is characterized by short and intense growing seasons with long daylengths, risk of early and late frosts, and generally lower disease pressure. The region is distinguished by its unique light qualities, which affect productivity and quality of the produce. A specific and economically important aspect of the Nordics are the wild crops and berries. These key characteristics of northern agriculture call for crops that are more climate resilient, perform well in northern latitudes, and are adapted to the agricultural systems in these conditions.

Human carbon emissions are elevating the concentration of CO_2_ in the atmosphere, amplifying the greenhouse effect, which leads to global warming and changes in precipitation. Climate change also increases the risks for adverse weather, inflicting production risks by causing variation in crop performance. Whereas many agricultural areas in the world will be negatively affected by global warming, there will be a prolonged growing season, making farming more profitable in the most northern agricultural areas. Consequently, the relative importance of agriculture in the Nordic countries is likely to increase in the future (Peltonen-Sainio and Niemi, 2012; Wiréhn, 2018). This also opens up the possibility for increased farming in the Arctic areas (see Box 1A). Climate change will affect the distribution of pathogens and pests, and the agriculture in the Nordic countries is predicted to be especially badly affected by invasive pests and increased plant disease pressure (Chaloner *et al.*, 2021). This calls for increased breeding efforts but also locally tailored measures in precision agriculture.

Box 1. **Arctic region in change**

Arctic agriculture refers to crop cultivation at high latitudes defined by the Arctic Circle. Most of the land areas above the Arctic Circle are currently locked in permafrost, expect the areas of northern Fennoscandia and parts of north-western Russia (Mølmann *et al.*, 2021a). Under the ongoing global warming, Arctic areas are predicted to warm up twice as fast as other areas and therefore potentially increase crop production in the upcoming years (King *et al.*, 2018; Altdorff *et al.*, 2021).

The Arctic regions have unique light conditions during the summer months including the midnight sun giving a 24 h photoperiod with an exceptionally long daily photosynthetic light period, together with characteristic diurnal spectral distributions depending on solar elevation. In high latitudes, the solar elevation angles are lower, which means that solar radiation has a longer path length through the atmospheric column, which influences the spectral light quality. For this reason, ozone selectively filters out more UV radiation at low solar angles and there is more effective scattering of blue compared with red light, leading to lower direct UV radiation and a higher proportional level of blue light in the daytime light spectrum. During the night-time hours, when the Sun angle is between 0° and 5°, the radiation at high latitudes is refracted and scattered through a longer atmospheric path, lowering the distribution towards far-red light (Nilsen, 1985; Mølmann *et al.*, 2021a).

The relatively low temperatures during the growth season together with the unique light conditions affect the growth, development, and quality of the crops produced at high latitudes (Mølmann *et al.*, 2021a). The predicted increase in temperatures, especially at the beginning of the growth season, will presumably extend the growth seasons and have a great effect on crop yields, and the selection of the crops that can be produced in these areas. However, increased precipitation and extreme weather episodes are potential challenges related to global warming affecting crop production in the northern regions. It also uncertain how the Arctic wild crops will adapt to the predicted fast changes to the climate.

Due to the predicted fast warming and the fact that ice is melting in the Arctic region, the EU has quite recently updated its Arctic strategy to obtain an understanding of the effects of climate change in these areas (European Commission, 2021). This includes the use of satellite imaging for following the development as well climate simulations. Preserving Arctic biodiversity is paramount for sustainable future of the region’s livelihood.

Breeding in the Nordic countries has until recently been characterized by low support for pre-breeding from their governments and seen as a niche market for international plant breeding companies (Nilsson and von Bothmer, 2010). This has hampered breeding of locally adapted crops. At the same time, recent heightened political instability and interruption to world trade in combination with larger demands for locally produced food (Niva *et al.*, 2014) have highlighted the need for increased production in the Nordic countries. To mitigate impacts of climate change and meet market demands, there is a drive to increase the adaptive capacity of crops. This should lead to resilient crops, which are those that are able to withstand various harsh and fluctuating growth conditions, and are suited to northern latitudes. It will require functional phenomics approaches that integrate high-throughput phenotyping (HTP), plant physiology, and bioinformatics (York, 2019). Functional phenomics seeks to fill the knowledge gap between plant phenotype and physiology by utilization of advanced sensor technologies and big data analytics.

In the wake of climate change and the increased need for food sovereignty, this review addresses three key objectives where phenotyping can advance and strengthen Nordic agriculture.

(i) Considering the specific Nordic conditions in plant breeding. By describing the current and future breeding needs for Nordic agriculture, we present key breeding traits that are or will be important to target. Examples of such traits are winter survival, tolerance to early spring drought, frost, and flooding, in addition to resistance to commonly occurring as well as emerging pests and pathogens. Functional phenomics should be applied to these specific targets to broaden the genetic resources used and increase the precision and genetic gains in breeding.
(ii) Maintaining plant health and improving crop management. To handle a changing climate, we highlight the need to better understand how current and future interactions between crops and pathogens and pests are manifested in the north. In addition, we highlight the need to explore the efficiency of biologicals and integrate functional phenomics in precision agriculture to further improve sustainable protection methods and more efficiently implement integrated pest management. We propose that this can be done by testing pathogenicity and host resistance at more northern latitudes and by comparative studies taking advantage of a latitudinal gradient.
(iii) Adopting and developing functional phenomics to meet Nordic agricultural needs. By describing the specific functional phenomics approach that relates phenotypes to function, we identify the particular needs and challenges for adapting this integrative, holistic approach to the current and future needs of Nordic agriculture. This complements the description of the present status of sensor-based methods available for controlled environments and outdoor phenotyping within the Nordic countries, and highlights the need to complement those with physiological approaches tailored to give a mechanistic understanding of the underlying processes for breeding and precision agriculture.

### The agricultural scene in the Nordic countries

**Fig. 1.**
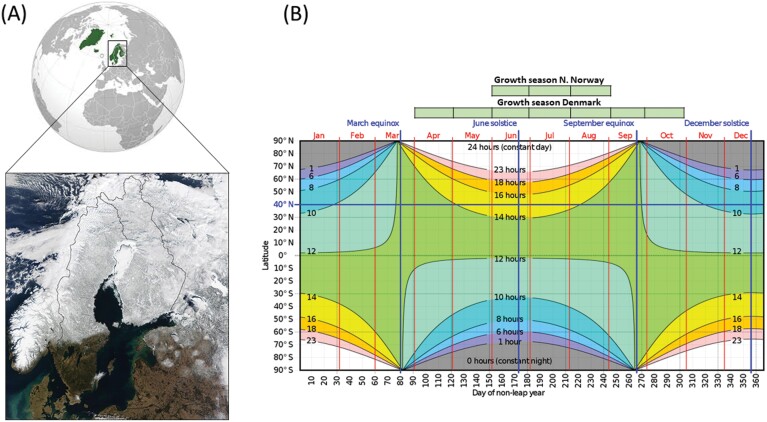
(A) The Nordic countries represents the most Northern agricultural areas in the world and the agriculture is characterized by short growing seasons with sometimes late frost and snow. Satellite image taken on 15 March 2002; (B) agriculture spans from 54° to 69° North, marked in red. The growth seasons in northern Norway and Denmark are marked in green boxes. Images from Wikimedia Commons (https://en.wikipedia.org/wiki/Nordic_countries#/media/File:Nordic_countries_orthographic.svg and https://en.wikipedia.org/wiki/Nordic_countries#/media/File:Nordic_countries_orthographic.svg, © public domain).

The five Nordic countries, Finland, Denmark, Iceland, Norway, and Sweden, are in the most northern growing region of field cultivation in the world, with a large latitudinal span from 54° to 69° north (Fig. 1). According to the Köppen–Geiger climate classification, the Nordic countries include four different climatic zones ranging from a warm temperate and warm-summer humid continental to subarctic, and polar tundra (Kottek *et al.*, 2006). The total crop production in these northern growing regions is relatively small compared with major production areas, and farming is often conducted in small units.

Within the next 50 years, the vegetation period is predicted to start 10–50 d earlier and end 5–50 d later depending on the Nordic region. According to the IPCC Sixth Assessment Report (https://www.ipcc.ch/report/ar6/wg1/), the average projected increase in temperature during spring, summer, and autumn, is 1–3 °C, with the greatest increase, even up to 6–8 °C, predicted in the northernmost areas. Main precipitation is estimated to increase up to 30% locally. The increase in the relative importance of Nordic agriculture is also a consequence of the fact that crop production in the traditional high-producing agricultural areas will be challenged by less favourable conditions with increased instances of drought and heat due to climate change. Despite the predicted positive impacts on the prolonged growth period, certain challenges are expected to increase in the future. For instance, simulations of future climate conditions show that due to the higher temperature and humidity, plant diseases will pose an increasing problem for Nordic agriculture (Chaloner *et al.*, 2021).

**Fig. 2.**
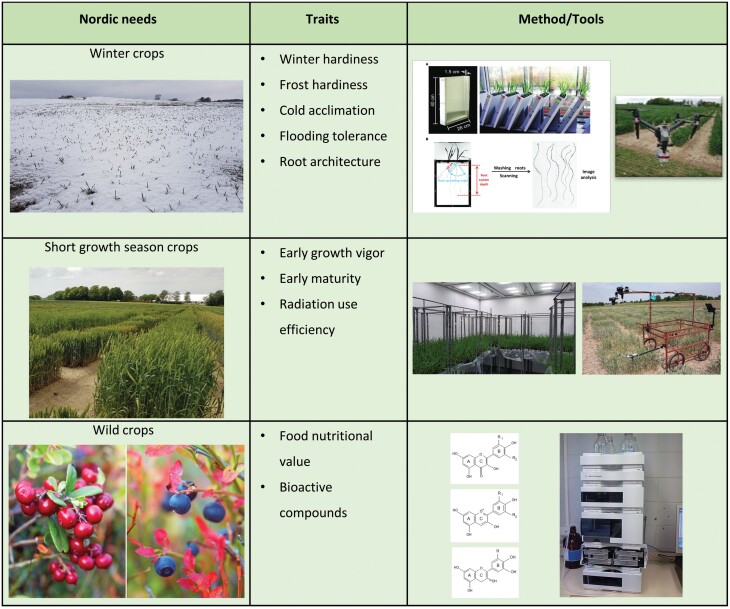
Three examples of the Nordic agricultural needs for plant functional phenomics. The corresponding target traits are shown together the methods or tools needed to study these. Lingonberry (*Vaccinium vitis-idaea*) and bilberry (*V. myrtillus*) are the two most common and economically most important wild berries in the Nordic countries. Both of these species are rich with flavonoid compounds, especially anthocyanins, flavonols, and flavan-3-ols (image sources: root phenotyping, Jia *et al.* (2019), HPLC image from Wikipedia (https://en.wikipedia.org/wiki/High-performance_liquid_chromatography#/media/File:Hplc.JPG, © public domain).

Agriculture production varies between Nordic regions, with barley, oats, and forage dominating in the north, whereas wheat and rye tend to be planted in the south together with potato, rapeseed, and sugar beet. Both Finland and Sweden are in the top 10 countries in the world in oat production, and Denmark is among the top 10 rye producers (FAOstat, 2021). With warmer climates, there has also been an increase in new crops such as maize. Maize is a C_4_ plant, which needs to be adapted to northern growing conditions, challenging the applicability of growth models for phenology and biomass (Jacobsen, 2017; Morel *et al.*, 2020). Other C_4_ crops such as quinoa might follow. There is also a renewed interest in protein crops, such as Faba bean and even soybean (Wiréhn, 2018; NordGen, 2019; Fogelberg and Mårtensson, 2021). In the Nordics, there is a high degree of organic farming, which calls for an alternative agriculture system and increased host resistance to reduce chemical pesticides and fertilizers (Pekala, 2020). A unique feature of the Nordics is the wild crops and wild berries that are the most important non-timber forest products (Box 2; Fig. 2) (Kottek *et al.*, 2006; Barua *et al.*, 2014; Chaloner *et al.*, 2021).

Box 2. **Uniqueness of wild crops**

Wild berries are the most important non-timber forest products (NTFP) or wild crops in the Nordic countries. It has been estimated that the yearly wild berry crop in the area of Nordic countries exceeds 1 billion kg, of which ~5–10% is utilized (Salo, 1995; Nestby *et al.*, 2019) even though the demand has been increasing. Wild berries are rich in bioactive compounds, especially vitamins, fibres, and phenolic secondary metabolites, such as anthocyanins and other flavonoids. Wild berry crop yields can vary dramatically between years, and this variation is linked to weather events. Therefore, it is important to determine the critical weather events affecting the quality of the crop and the fluctuation in the crop yields for estimation of the effect of global warming (Nestby *et al.*, 2019). A temperature-controlled study with bilberry (*Vaccinium myrtillus* L.) reported higher anthocyanin levels and lower sugar levels at higher temperature (18 °C) compared with lower temperature (+12°C) growth conditions (Uleberg *et al.*, 2012). However, southern and northern ecotypes showed different responses, which indicates a need for more in-depth studies on understanding the adaptation of wild plants in their local growth conditions.

The Nordic labour market is characterized by high levels of employment, education, and salary, even for unskilled labour. Despite the high labour costs and unfavourable climate, southern agricultural regions of the Nordics have high labour productivity (Giannakis and Bruggeman, 2018). To give Nordic agriculture added value, focus will probably be on nutrient content rather than crop yield. Nordic agriculture could play a role in the important shift towards producing more plant-based proteins and nutritious crops to counteract the current global imbalance where there is an overproduction of starch and sugars compared with nutritional recommendations for human food intake (Kc *et al.*, 2018). Moreover, there is a drive to increase productivity through automation and a technological shift of which plant phenotyping will be an integral part.

Taken together, there is an urgent need to better understand crop performance in Nordic climate conditions and future climate scenarios, to support the increasing importance of Nordic agriculture by addressing the three objectives outlined above. An efficient and targeted phenotyping of traits important for Nordic agriculture (Table 1; Fig. 2) will be an essential cornerstone in the adaptation process, which needs to be linked to the plant function. As pointed out by Rosenqvist *et al.* (2019), plant phenotyping has gradually shifted from basic to strategic crop research linked to classical agricultural traits. Although this review focuses on the Nordic region, the ­content also applies to other northern growing areas such as the Baltic Sea region, Northern Russian, and Canada.

**Table 1.** Challenges the Nordic agriculture is facing currently, and their future predicted impact due to climate change

Challenges for Nordic agriculture	Future predicted impact	Traits to target	Phenotyping needs	Publications with Nordic perspectives or phenotyping solutions
**Abiotic**				
Cold winters	↓	Winter hardiness	Climate and freezing chambers <0 °C, robust field phenotyping equipment, root phenotyping, stomatal conductance	Chawade *et al.* (2012); Alexandersson *et al.* (2018); Michel *et al.* (2019)
Frost early and late during the season	→ ↑	Frost tolerance	Climate and freezing chambers <0 °C, robust field phenotyping equipment,stomatal conductance	Lorente *et al.* (2012); Michel *et al.* (2019)
Short growing season	↓	Development	Time series phenotyping in controlled and field conditions, root phenotyping	Borner *et al.* (2008); Econopouly *et al.* (2019)
Micro-droughts	↑	Drought tolerance	Irrigation-controlled, controlled, and field phenotyping, root phenotyping	Kumar *et al.* (2020)
Flooding	↑	Flooding tolerance	Irrigation-controlled, controlled, and field phenotyping, root phenotyping	Mustroph (2018)
Heat waves	↑	Heat tolerance	Climate chambers with programmable fluctuating climate parameters	Chawade *et al.* (2018); Abdelhakim *et al.* (2021)
Light composition/light intensity	→	Leaf properties, photosynthesis, radiation use efficiency	Programmable LED light chambers, photosynthetic rate measurements,latitudinal studies in crop fields	Gspaltl *et al.* (2013); Dusenge *et al.* (2019); Mølmann *et al.* (2021b)
**Biotic**				
Invasive pathogens and pests due to climate change	↑	Resistance mechanisms,disease progression and severity indicators	Pathosystems adapted to high-throughput imaging platforms, enzyme activity signatures, access to field phenotyping stations on mainland Europe, latitudinal studies of crop fields, spore and insect traps to characterize disease pressure	Chawade *et al.* (2016); Odilbekov et al. (2018, 2019); Alemu *et al.* (2021); Gao *et al.*, (2021); Pavicic *et al.* (2021)
**Other challenges**				
Yield	↑	Growth rate,yield factors, photosynthetic rate	3D phenotyping of biomass, access to field phenotyping stations for latitudinal studies	Haverkort and Verhagen (2008); Göransson *et al.* (2019); Kumar *et al.* (2020); Theeuwen *et al.* (2022)
Food nutritional value	↑	Bioactive compounds	Combination of analytical chemistry and enzyme activity signatures, climate chambers, spectral quality treatments	Uleberg *et al.* (2012); Samkumar *et al.* (2021)
New consumer demands such as new Nordic diet	↑	Yield traits in minor and local crops	Broad phenotyping of genetic resources including Nordic landraces, domestication of wild species	Kaur *et al.* (2020); Kumar *et al.* (2020); Liu *et al.* (2021)
Small farms	↓→	Yield traits for high-value crops	Affordable phenotyping solutions to lower the phenotyping cost barriers for minor crops	Kaur *et al.* (2020); Liu *et al.* (2021)
Sustainable intensification	↑	Growth and performance	Phenotyping plant growth and performance in response to changing management procedures—such as reduced use of fertilizers and pesticides or application of biostimulants	Voss-Fels *et al.* (2019); Rouphael and Colla (2020)
Phenological responses, such as flowering time induction during mixed autumn conditions	↑	Flowering time determination, high-value fruit crops and trees	Detection of increased IR (750 –900 nm) reflection in buds or RGB image-based analysis of developmental timing of flower induction	Zhang *et al.* (2015); Forsström *et al.* (2019); Linkosalo (2019)
Early vigour	↑	Germination rate, soil coverage rate	Detection of germination and early growth vigour by RGB or chlorophyll fluorescence in temperature treatments	De Diego *et al.* (2017); Pavicic *et al.* (2019)
Maturation time	↓	Growth, developmental timing, photosynthesis, yield	Developmental timing of flowering or yield factor detection by RGB or chlorophyll fluorescence imaging methods in higher temperatures and higher CO_2_ level conditions	Baslam *et al.* (2020); Theeuwen *et al.* (2022)

The aim is to define traits that could be assessed by phenotyping and functional phenomics. Arrows pointing down indicate reduced impact due to climate change; arrows pointing up indicate increasing impact due to climate change; and arrows pointing to the side indicate no change in impact due to climate change in a Nordic perspective.

## Breeding for future Nordic climates

Key traits in Nordic agriculture required to obtain higher or acceptable yields are winter hardiness in winter crops, frost tolerance, drought tolerance, early vigour, maturity time, lodging tolerance, and resistance to important diseases. Breeding new varieties for higher yield, wider adaptability, and stable production across different regions has traditionally been the goal of plant breeding all around the world (Nehe *et al.*, 2019). There are limited recent studies in the Nordic region on cultivated crops and plants where the genotype × environment interaction (GEI) was studied. In addition, very limited initiatives have been taken to use HTP indoor facilities or controlled-climate chambers to accelerate breeding for Nordic conditions. However, there are increasing efforts to use advanced sensors and automated phenotyping systems in the field through different public–private partnership projects. In spring barley and wheat, for example, studies are underway using HTP indoor facilities targeting Nordic growth conditions for early vigour and drought, respectively, as part of the EPPN2020 initiative.

### Diverse cultivation environments

Amongst the crops, forage perennial grasses and legumes are grown on a larger scale than any other crop in Nordic countries. As they are growing for a longer duration compared with seasonal crops, forage crops will be more affected by adverse growing conditions and climate change. Among food crops, spring barley is central in the Nordic countries where yield performance depends on GEI. For example, six-row barley performed better than two-row barley in the northern than in the southern part of the Nordic growing region (Nurminiemi *et al.*, 2002; Hilmarsson and Rio, 2021), and southern regions are more productive than northern regions (Nurminiemi and Rognli, 1996). This GEI can be explained by the higher mean temperature in the southern part, and the consequent earlier flowering and maturity that allow faster growth following the principle of thermal time. Therefore, regarding climate change in the near future, heat stress could be more serious than drought stress. In that case, breeding strategies could be focused on developing heat-tolerant genotypes.

Only very limited information is available on how climate change will affect adaptability and phenotypic stability in minority crops. To relate the observed pattern of adaptation to the geographic origin of the cultivars and their response to prevailing agroclimatic conditions, Helgadóttir *et al.* (2018) compared the potential of biomass production in *Lolium perenne* L. cultivars of diverse origin across five locations stretching from Estonia to Iceland. A recent study conducted over 3 years in eight different northerly field locations concluded that the genepool of Nordic spring barley still harboured allele combinations suitable for local adaptation and further expansion of cultivation to the north (Göransson *et al.*, 2019). Moreover, breeding strategies in the north should focus on radiation use efficiency (RUE) as both light and temperature are limited. Historical radiation data are available in Sweden, but they have not been exploited for crop breeding (Perttu and Morén, 1994; Eckersten *et al.*, 2008). Multilocation trials characterizing different genotypes for traits such as early vigour, flowering, maturity, increasing sink strength, and photosynthetic efficiency will be key to developing high-yielding cultivars for Nordic climates. Other traits such as lodging resistance in wheat are important in countries such as Iceland (Bragason, 1985), and in the future this trait could be more relevant as extreme weather events may lead to higher wind speed resulting in more lodging (Skov Kristensen *et al.*, 2016).

### Emerging abiotic stresses

The changing climate is expected to lead to reduced soil moisture availability in the top soil during the peak growing season (Nehe *et al.*, 2021). This can lead to drought-related reductions in yield and quality in various crop species. Warmer winters can also lead to reduced snow cover, leading to reduced soil water availability in spring (Trnka *et al.*, 2021). Late spring drought is an emerging challenge in Nordic conditions, and it needs to be addressed in almost all spring-sown crops. It is a relatively greater concern in spring-sown crops as it can cause damage in plants that are not yet well established (Tao *et al.*, 2015; Chawade *et al.*, 2018). Late summer drought is also becoming a great concern as during the last years it has been observed relatively frequently in some regions of Northern Europe and especially in Southern Sweden and Denmark. An extreme drought period was observed in 2018 leading to significant loss in yield and quality of the produce (Kumar *et al.*, 2020). In a winter wheat trial conducted in 2018, genotypes from the Nordic genebank NordGen were characterized in the field for drought tolerance by proximal phenotyping using consumer-grade cameras mounted on a phenocart (Kumar *et al.*, 2020).

### Temporal resolution for phenotyping

Changing climate is expected to lead to longer growing seasons and the achievement of higher grain yields and protein quality in cereal crops if certain breeding traits such as time of anthesis and rate of grain filling can be optimized (Semenov *et al.*, 2014; Nehe *et al.*, 2020). These optimizations can, however, differ based on in which Nordic region the crop is to be grown. While in central and southern Sweden and Denmark later heading is desired, in the more northern parts, early heading is more desirable due to a relatively shorter growing season. Thus, field and indoor phenotyping is commonly used, as automated phenotyping allows for continuous phenotyping of plants, providing a detailed analysis of plant growth over time. Image-based sensor technologies allow the generation of temporally highly resolved phenotyping data for analysis of growth and physiological responses (Pavicic *et al.*, 2017). Higher temporal resolution has the advantage that it not only records a given plant trait at a certain stage, but also enables the evaluation of how quickly the trait develops, and how soon the trait reaches its maximum or desired range. This allows for comparative analyses of a given trait and possibly selection of superior genotypes with much higher precision. Yet another benefit from regular phenotyping is the information obtained on how the trait interacts with and is affected by abiotic and biotic factors observed in the field over a given time period. For instance, a sudden onset of late spring drought can be manifested through changes in the dynamic growth patterns wherein certain genotypes might show reduced growth due to the sudden onset of drought. This allows for a high-precision evaluation of not just the trait but also occurrences of various environmental changes during the life cycle of the plant. Dynamic growth patterns are commonly estimated for plant growth (biomass), plant height, and maturity (Nehe *et al.*, 2020). Additional traits that could benefit from higher temporal resolution are counting of organs such as number of flowers or fruits over time.

### Phenotyping of quantitative traits

Winter hardiness is difficult to phenotype not least due to its irregular occurrences in field trials and high cost to screen in controlled environments (Chawade et al., 2012, 2013; Lindlöf *et al.*, 2015). Furthermore, scoring for winter hardiness for the autumn-sown crops in early spring can be challenging as the early vigour trait could be mistakenly identified as winter survival. This could be especially challenging with drone-based phenotyping, and thus the analysis pipeline should be able to differentiate the early vigour trait from winter survival. Michel *et al.* (2019) found a high level of heritability (*h*^2^=0.98) for frost tolerance under controlled conditions in wheat. Image-based methods have also been used to monitor Canadian winter wheat performance. Y. Chen *et al.* (2019) compared different monitoring methods of performance including unmanned aerial vehicles (UAVs) with multispectral imaging in field trials and could show that images captured by UAVs provided a good estimation of damage compared with other methods of capture. With appropriate UAV flights and image analysis, plant height measured by UAVs can have a high correlation with manually measured height. Correlation between UAV and manually measured plant height was as high as 0.91 in a study of wheat breeding lines (Volpato *et al.*, 2021). Several traits can also be reliably measured using imaging under controlled and field conditions, including drought stress and early vigour in wheat (Armoniené *et al.*, 2018; Kumar *et al.*, 2020).

### Benefits of the tradition of public–private partnerships in Nordic breeding

Food security relies on the resilience of staple food crops to climatic variability and extremes. In Nordic countries there is a long tradition of private–public breeding partnerships compared with central European countries such as Germany, France, and Spain (Kahiluoto *et al.*, 2019). The specific Nordic challenges for breeding and the need for phenotyping have also been recognized by agencies that support regional networking. The established networks including public–private partnership projects bring together universities and companies to share plant phenotyping research facilities and help in integrating phenotyping methods into breeders’ daily activities. There are still few phenomics facilities combining HTP and advanced climate simulation, but these will be needed to facilitate pre-breeding efforts for better adaption of crops to the Nordic conditions and for future climate scenarios (Alexandersson *et al.*, 2018).

## Plant health and crop management in Nordic conditions

Traditionally Nordic agriculture has been characterized by a low degree of crop disease because of the unfavourable winter conditions for pathogens and pests. Increasing temperatures, altered water availability, especially increasing humidity, and elevated levels of CO_2_ are predicted to increase the incidences of crop diseases in the Nordic region. These environmental factors will influence both the geographical distribution of pests and pathogens and host resistance, since the plants’ physiology and the innate immune system are affected by the altered growth conditions. Chaloner *et al.* (2021) recently presented a climate model including 80 fungi and oomycete crop pathogens in 12 crops. This model showed that even if agriculture production is expected to increase at high latitudes, an increase in disease load is also predicted. The increased disease pressure due to warmer climates and longer seasons will hamper production. To this end, plant disease phenotyping will be important to evaluate crop germplasms and to find appropriate management systems. Both disease surveillance in the field and disease resistance phenotyping for breeding will be important tasks within functional phenomics.

### Phenotyping for resistance breeding and disease surveillance

The use of herbicides against weeds is relatively high in the Nordic countries. Automated detection of weeds by various computer vision methods will be of great help in future Nordic precision agriculture and could reduce the dependence on herbicides. For example, Leminen Madsen *et al.* (2020) developed an open plant phenotype database based on the natural occurrence of 48 weed species and their importance for arable crop farming in Denmark.

There is a lack of a coherent monitoring system of weeds and diseases, and a need for regional early-warning systems as plant health challenges follow climatic zones and agricultural practices rather than national borders (Fears *et al.*, 2014). Satellite phenotyping or phenotyping at the field level for reporting could advance disease surveillance but still needs to be developed, and the importance of image- and sensor-based phenotyping for surveillance in agriculture still has to be further evaluated (Chawade *et al.*, 2019). In forestry, there has been some success in monitoring pests in Nordic countries (e.g. Olsson *et al.*, 2016; Huo *et al.*, 2021).

Image-based phenotyping of disease processes in time series can help to study both climate-dependent and genotype-dependent disease progression and severity (Pavicic *et al.*, 2021). Automated phenotyping with the help of UAVs or robotics can increase the number of measuring times compared with manual handling by traditional scoring based on visual inspection. Some pathogen infections only become visible by the naked eye at later stages of plant disease development, but can instead be captured pre-symptomatically by multispectral, hyperspectral, multicolour fluorescence, and PAM chlorophyll fluorescence imaging (Hupp *et al.*, 2019; Pandey *et al.*, 2021). In spite of these emerging possibilities, there are so far relatively few studies in agricultural fields of disease progression using proximal or remote sensing in Nordic conditions, and a functional phenomics approach has not been taken. However, proximal and UAV-based sensing of *Septoria tritici* wheat blotch disease and potato late blight have been done in field ­conditions in Denmark and Sweden, respectively (Odilbekov *et al.*, 2018; Gao *et al.*, 2021).

### Phenotyping along the latitudinal north–south gradient

The presence of plant pests and pathogens is following geographical patterns. In ecology, several hypotheses have been put forward based on this trophic interaction, which is also thought to follow a latitudinal gradient with increased interactions towards lower latitudes (Coley and Aide, 1991; Schemske *et al.*, 2009). The latitudinal variance seen in plant defence is shaped by many factors, including environmental factors such as light quality. Comparative studies including Nordic conditions are interesting because of the specific interactions between light and temperature cycles that are known to affect pathogen inoculation and infection progress. Concerns over the lack of measuring different types of plant defence responses as well as the measurement of too simplistic proxies of defence responses in these studies have, however, been raised (Anstett *et al.*, 2016). To this end, different vegetative indices determined by remote sensing could be helpful to include as a measure of the health status of crops at the field or even the landscape scale. North–south comparisons of vegetative indices related to plant health could help in understanding latitudinal effects of plant defence. Such latitudinal studies could be specifically important for Nordic agriculture. A few studies, mostly in Arabidopsis, have investigated the relationship between ecotypes from different latitudes and host defence (Todesco *et al.*, 2010). Nonetheless, these types of studies, which could help to better understand local defence adaption mechanisms, have not yet been connected to agricultural traits or been subjected to HTP. In general, there are more latitudinal studies on the spread, damage, and variance in resistance against herbivores than for microbe plant pathogens, which is a study area that should be expanded. To improve crop resilience, traits measurable for herbivore tolerance have been suggested (Mitchell *et al.*, 2016). A better exploration of the concept of plant host tolerance in the Nordics should be carried out, since it holds promise in future organic farming, which is of significant interest for the local food market.

### The effect of current and future Nordic climate on host resistance and specialized metabolites

Over the past years there has been more evidence that light conditions have a strong effect on plant defence (Roberts and Paul, 2006), including the strong connection between the availability of resources for primary metabolism and for plant responses to pests and pathogens (Bolton, 2009). Because of increased heat, molecular mechanisms and genetic factors have been linked to defective growth, temperature-dependent autoimmunity, and other effects on immune signalling (Venkatesh and Kang, 2019).

Primary defence mechanisms of plants are based on the hydrophobic cuticular surface of the aerial parts and the various plant secondary metabolites. The cuticular layer is composed of a polyester mixture of lipidic compounds collectively called wax, the composition of which varies between species and is affected by environmental conditions (Trivedi *et al.*, 2019). Plant secondary metabolites found in the cuticule and throughout all tissues and organs offer protection against pathogens and pests, and presumably the wide variation of these compounds has evolved as a resistance mechanism in plants (Kessler, 2015). It is well documented that light conditions affect biosynthesis and accumulation of secondary metabolites in plants, both the spectral composition of light and its intensity, but also the daily periods of light (Zoratti *et al.*, 2014; Thoma *et al.*, 2020), something to be studied in more depth in Nordic conditions.

For food production, a special concern are toxins produced by plant pathogens, which might become an increased concern. For example, with warmer and more humid conditions Fusarium head blight infections on cereals are increasing in northern Europe (Parikka *et al.*, 2012), leading to increased occurrence of *Fusarium* mycotoxins. However, there are breeding efforts including genomic selection in, for example, Nordic oats to increase host resistance and thus reduce toxin levels (Haikka *et al.*, 2020). Concerns have previously been raised about *Alternaria* toxins in tropical fruit production and in blueberry production in colder climates of Argentina, but these remain unstudied in European conditions (Greco *et al.*, 2012).

Another climate effect is increased herbivory (Deutsch *et al.*, 2018). Apart from direct yield losses, herbivory induces the production of volatile organic compounds (VOCs), which have a crucial effect on the amount of carbon plants fix, as they represent close to 10% of photosynthetically bound carbon and consequently the amount of carbon released to the atmosphere (Kesselmeier *et al.*, 2002). Using open-top chambers, Li *et al.* (2019) recently showed that an increased temperature increased herbivory as well as the amount and composition of VOCs emitted from birch trees. Still, estimates of VOC fluxes at large scales and how they act in a warmer world with elevated CO_2_ levels are associated with great uncertainties (Jiang *et al.*, 2019). The combination of plant phenotyping and experimental set-ups to measure gas exchange in the interaction of higher temperatures, specific light conditions, and plant VOCs should be developed in Nordic and Arctic conditions to further assess the future contribution of greenhouse gases.

### Exploring Nordic microbiomes and Nordic holobiont physiology

Plants, like all multicellular organisms, are colonized by microbes, and it is believed that they have outsourced several key functions to their microbial partners—the so-called microbiomes (Mendes and Raaijmakers, 2015). Deciphering how interactions between microbes, plants, and soil confer improved resistance of the host plant to abiotic and biotic stress, or impact plant nutrient acquisition under current or predicted climate conditions, is an enormous challenge (Trnka *et al.*, 2014). The determination of bulk soil microbiomes along a 1650 km climatic gradient through the maritime Antarctic revealed that the expected further rises in air temperature in the maritime Antarctic may enhance terrestrial ecosystem productivity through positive effects on soil bacterial diversity (Dennis *et al.*, 2019). With a few exceptions of studies of tree microbiomes (Bálint *et al.*, 2015; Habiyaremye *et al.*, 2021), this is notably unexplored under Nordic conditions. The influence of the specific Nordic light conditions should also be explored further as light quality is known to influence plant–microbe interactions (Alsanius *et al.*, 2019). Hence, it is now time to significantly advance research within this area to the next level, where microbiomes are not only described as collections of microbes colonizing a host. Instead, we propose a holistic perspective to describe a complex holobiont entity, emerging as the result of myriads of interactions between the plants and their associated microbes (Garcia-Lemos *et al.*, 2019). Again, latitudinal studies focusing on a north–south transect should be useful.

### Adapting biologicals to the Nordic conditions

Collectively, biostimulants and biocontrols are known as biologicals, which can give crops good baseline strength and health, making them less vulnerable to stress, pests, and other threats, including climate shocks. Biologicals can help farmers adapt their agricultural systems to an increasingly volatile climate while enhancing food production sustainability, thereby contributing to a climate-smart farm and environmentally friendly model for the future, which is both resilient and flexible.

Moreover, even if there is an urgent call to reduce the usage of chemical pesticides, there are few studies on the effect of Nordic climates on the effectiveness and durability of biologicals. There is a direct lack of comparative latitudinal studies on the effect of application of biologicals in spite of their potential in mitigating abiotic and biotic stresses (Sandroni *et al.*, 2020; Del Buono, 2021). In combination with a deeper understanding of the mode of action of biologicals in plants, defence responses triggered by elicitors in different climate conditions could also be assessed. Climate and phenomics facilities could be used for efficient screening (Rouphael *et al.*, 2018) to further develop biostimulants for agriculture (Rouphael and Colla, 2020) also for Nordic conditions.

## Functional phenomics and phenotyping facilities

### Application of functional phenomics within a Nordic context

Unlike conventional quantitative genetics, integrative functional phenomics involves the detailed analyses of physiological parameters, considers the underlying processes, their genetic basis, as well as environmental impacts and agricultural practices, in combination with complementary non-invasive sensing and imaging techniques (Furbank and Tester, 2011). This concept is valuable in particular for certain field applications because complex crop phenotypes with quantitative features and their response to the environment cannot be understood and predicted based solely on the genes specific to a single macroscopic phenotype *per se* (Wollenweber *et al.*, 2005). In fact, large-scale molecular data (so-called ‘-omics’ data) obtained in field trials combined with phenotyping opens up new possibilities to understand the molecular and environmental complexity of the agricultural field environment (Alexandersson *et al.*, 2014). Armed with this systems biology, interdisciplinary approach, plant physiology and functional genomics complement each other, ultimately enabling the *in silico* assessment of responses to genetic fine-tuning under defined environments. The physiological and molecular data will be verified by functional approaches and applied to improve crop plants to increase harvest yield and their stress tolerance, and to use them as bioreactors for high-value products. However, the lack of systematic multiyear and multilocation field trials analysed by multiomics techniques in Nordic countries and notably along climatic transects so far has hampered the broad application of a functional phenomics approach.

### Integration of physiological phenotyping to asses genome×environment×management interactions

Changing climate conditions will require region-specific breeding targets as climate change is expected to impact differently even within the Nordic region. Crop plant phenotypic plasticity is a result of the complex responses to GEI, with an additional input from management interventions. Whereas great advances have been made in the cost-efficient and high-throughput analyses of genetic information and non-invasive phenotyping, large-scale analyses of the underlying physiological mechanisms lag behind. The external phenotype is determined by the sum of the complex interactions of metabolic pathways and intracellular regulatory networks that is reflected in an internal, physiological, and biochemical phenotype. These various scales of dynamic physiological responses need to be considered, and genotyping and external phenotyping should be linked to the physiology at the cellular and tissue level. A high dimensional physiological phenotyping across scales is needed that integrates the precise characterization of the internal phenotype into HTP of whole plants and canopies (Großkinsky et al., 2015, 2018). The determination of enzyme activity signatures is emerging as a robust, semi-high-throughput tool to bridge the genotype–phenotype gap to assess the impact of pedoclimatic condition and the integration of environmental variables and crop management in the genetic programmes (Gibon *et al.*, 2004; Jammer *et al.*, 2015; Fimognari *et al.*, 2020), and is also reviewed in this special issue (Jammer *et al.*, 2022). By this means, complex traits can be broken down into individual components of physiological traits. Since the higher resolution of physiological phenotyping by ‘wet chemistry’ will remain inherently limited in throughput, non-invasive HTP needs to be validated and verified across scales to be used as proxy for the underlying processes. Such deep physiological phenotyping is so far lacking for Nordic climates.

### Nordic controlled-climate growth and high-throughput phenotyping facilities

**Fig. 3.**
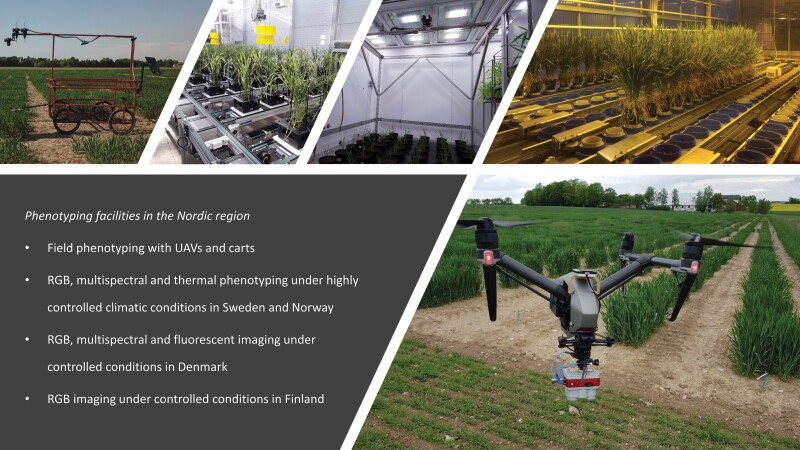
Examples of phenotyping facilities in the Nordic region.

Major changes in growing conditions are predicted for Nordic agriculture. Notably, the predicted changes in temperatures will impact growing seasons and thereby potentially plant phenologies (Hildén *et al.*, 2005; Borner *et al.*, 2008). It is challenging to predict plant phenological responses, but the climate models and predicted climate conditions can be combined with germplasm screening programmes to facilitate these and strengthen climate-resilient ‘Nordic traits’ (Table 1; Figs 2, 3). Climate facilities that allow testing of expected future environmental conditions in combination with the particular Nordic abiotic factors, their combinations, and gradients, together with HTP of crop responses, can greatly facilitate screening for improved climate resilience in germplasm collections. Although the majority of phenotyping facilities are in controlled climates, there is a lack of facilities combining HTP with highly precise and adjustable climate simulations (Yang *et al.*, 2020). The specific Nordic weather conditions would, for example, require phenotyping facilities that can simulate temperature gradients in combination with the specific light intensities and qualities. In Sweden, in the controlled-climate growth facility called Biotron, a new phenotyping system, Phenocave, was recently established (Leiva *et al.*, 2021). Phenocave supports fully automated RGB, multispectral, and thermal imaging. As Phenocave is established within the controlled-climate chambers, a wide range of climatic conditions for plant growth are possible along with automated sensor-based phenotyping. In Finland, the national plant phenotyping infrastructure (NaPPI) allows phenotyping of small and large crop plants in automated imaging facilities for growth, development, and physiology in either controlled or monitored environments and management regimes. In addition, NaPPI allows assessment of the impact of light quality, such as far-red and red light supplements during gradual dusk and dawn illumination in different daylength regimes (Alexandersson *et al.*, 2018). The Climate Laboratory in Tromsø is jointly maintained by The Arctic University of Norway (UiT) and the Norwegian Institute of Bioeconomy (NIBIO). Being located at 69°39ʹN, 350 km north of the Arctic Circle, it is the northernmost phytotron in the world and offers special opportunities to study growth with the unique light ­conditions characteristic of the Arctic. During the summer there is practically no dark period, and the spectral light composition is distinctly different from that at lower latitudes. The Climate Laboratory has recently acquired flexible phenotyping instruments including a Planteye multispectral 3D scanner (Phenospex, Heerlen, The Netherlands) and hyperspectral camera systems ideal for use in controlled greenhouse conditions. The Centre for Plant Research in Controlled Climate (SKP), in Ås, South Norway, a joint unit between the NMBU (Norwegian University of Life Sciences) and NIBIO, also offers a climate-controlled facility for research purposes. Automated HTP in the PhenoLab at the University of Copenhagen allows assessment of the impact of various abiotic environmental factors including expected future CO_2_ levels on plant responses by multireflectance, fluorescence, and thermography (Pandey *et al.*, 2021). Finally, the Umeå Plant Science Centre hosts a unique conveyor belt-driven tree phenotyping platform which automatically monitors and records growth parameters.

An increasing major challenge in phenotyping is the great number and heterogeneity of data obtained with diverse methods that need to be handled and integrated (Pieruschka and Schurr, 2019). In particular, the implementation of multiscale physiological phenotyping, which includes the association of phenotypic information with a multitude of metadata and integration of highly diverse datasets, such as data deriving from genomic or biochemical analyses, will further contribute to the complexity related to acquisition, handling, and analysis. Improved data management approaches will allow the capture of the comprehensive view of the plant performance and responses. We foresee future efforts to more systematically record and integrate additional -omics data into phenotyping. Here we see a need also to collect and organize phenotyping and -omics data into the Nordic field trial database.

### Root phenotyping

There is still little attention paid to the importance of root phenotyping in general which notably also applies to research for Nordic agriculture. Both above- and belowground phenotyping under Nordic field conditions will be important to measure crop biomass to assess the effect of biostimulants and the holobiont physiology, as well as carbon sequestration. Improved and automated root phenotyping will be relevant for winter hardiness and early vigour of the important Nordic breeding targets. So far there are two unique semi-field facilities, both in Denmark, to study roots in a Nordic environment: the Radimax facility enables large-scale screening for deep rooting in combination with direct root phenotyping (Svane *et al.*, 2019), and the Rhizobox laboratory enables detailed studies of root growth and function, by having access to soil and roots up to a depth of 4 m (Thorup-Kristensen *et al.*, 2020). Within the Radimax facility, roots are monitored *in situ* by multireflectance imaging. The user-friendly convolutional neural networks solution RootPainter has been developed for faster and more accurate root image analysis, thus significantly reducing the time required for root measurement (Han *et al.*, 2021). With these approaches, root traits for deep nitrate uptake under Nordic conditions have been identified (Wacker *et al.*, 2022). Such facilities could be used to test root establishment and uptake of nitrogen in cereals, which can be important to screen for winter varieties and vigour in general ( S. Chen *et al.*, 2019). Estimation of root biomass and depth can help in breeding of more drought-tolerant crops with increased nutrient uptake. Furthermore, a larger root system can increase the carbon sequestration potential and mitigate elevated CO_2_ levels (Kell, 2011). There is, however, a clear need for an advanced facility in the northern part of the Nordic agricultural region, as this would give a better understanding of root development and architecture in more extreme conditions. In addition to the identification of new adapted cultivars, adjustments of management practices such as sowing time might be required to better match the new climate conditions. In general, there is a need for HTP and precise phenotyping efforts evaluating the impact of different management practices.

Combining functional phenomics and root economics was shown to be a promising approach to improve the understanding of crop ecophysiology (Guo *et al.*, 2021). The finding that HTP of respiration and architecture in wheat made it possible to identify root traits and genomic regions that could be harnessed to breed more efficient crops for sustainable agroecosystems could serve as a model for corresponding approaches in the Nordics.

### Phenotyping of field crops and wild crops

The Nordic environment also provides a unique resource of wild crops which are rich in bioactive compounds but that need to be studied in more detail using functional phenomics. These studies can help in the understanding of the benefits of the Nordic diet and what type of environments influence these compounds, and also make them available for more consumers, thus contributing to improved health and economy.

Advanced image-based phenotyping will also be needed for the estimation of the adaptation of the wild crops to changing climate conditions. For this, remote sensing methods using aerial phenotyping platforms including satellites and UAVs (drones), widely used in field phenotyping, are the most suitable systems. Satellite images can be used for quantification of biomass variation in given areas, but the number of cloud-free days in temperate regions such as the Nordics is a limitation, together with lower image resolution and tree canopies covering the wild crops (Söderström *et al.*, 2016; Song *et al.*, 2021). Therefore, UAVs could be preferable platforms for the wild crops, especially to be used in certain areas with easy access and for analysing phenological series of the same plots. Advantages with UAVs are flexibility and the potential to produce high spatial resolution images and the use of multi-imaging sensors, such as visible light (RGB) and multispectral cameras, infrared thermal imagers, LiDAR and hyperspectral cameras (Roitsch *et al.*, 2019; Song *et al.*, 2021). Narrow band reflectance measurements have been used for estimating the growth, ­flowering, fruit set, and crop yields for the wild blueberry fields in northern America (Percival *et al.*, 2012). The dense forest canopies, however, can create some challenges for the wild berry crops in the southern parts of the Nordic countries. The use of UAVs will require ground truthing with high precision field phenotyping approaches such as the PhenoField developed at the University of Copenhagen (Svensgaard *et al.*, 2014).

## Conclusion and future perspectives

Currently, farming in the far north is a small part of global agriculture production. However, with the ongoing global warming leading to prolonged and more favourable growth seasons, it will play a proportionally more important role contributing to a larger part of global agricultural production. Still, Nordic agriculture requires farming in extreme environments and, for it to make a substantial contribution, a number of technological tools are needed to increase the speed of crop adaptation and to provide efficient production systems. Together with appropriate genetic resources, plant phenotyping integrated into a functional phenomics approach is at the very core of these and can increase the selection intensity in breeding programmes.

Therefore, more efficient use and development of the existent HTP and controlled-climate growth facilities combined with flexible phenotyping systems offer possibilities for screening of suitable plant material for Nordic conditions and improving aspects of future plant health. This includes developing root phenotyping prospects further north, as pointed out in this review. Functional phenomics should also be used to assess the possible future contribution of Nordic and even Arctic agriculture as a carbon source–sink in an effort to mitigate elevated CO_2_ levels. Better availability and cooperation between Nordic phenotyping facilities would increase the ability to characterize plant genetic resources in collaboration with the NordGen genebank. In addition, comparative and interdisciplinary latitudinal field studies using advanced phenotyping could contribute to better understanding of crop performance and resilience, by adopting knowledge from ecological studies describing latitudinal effects. At the same time, a decreased cost of consumer-grade phenotyping equipment will lower the cost barrier for breeding for niche crops for the smaller Nordic market.

An increased focus on functional phenomics has the potential to support Nordic breeding of horticultural crops, which during the last 20–30 years has been lagging behind. This is especially important since horticultural crops are an important part of the value chain in Nordic farming, and the current increase in consumer demands of local produce. In addition, crop phenotyping can be a part of decision support systems for precision agriculture in Nordic farming to contribute to reduction of inorganic fertilizer and agrochemicals. We believe that further development in this area would benefit from ­public–private partnership similar to the tradition in pre-breeding in the Nordic countries.

The successful Nordic public–private pre-breeding projects, to channel genetic resources into plant breeding and to support Nordic plant breeding efforts, need to be strengthened and further developed by incorporation of functional phenomics. The relatively small but cross-disciplinary Nordic phenotyping community can help to achieve these goals by developing both high-tech solutions for academic research and affordable, flexible, and user-friendly phenotyping solutions tailored to the practical needs for breeding and precision agriculture to ensure that the Nordics will also be part of the second green revolution.
